# Retrospective Study of Functional and Esthetic Outcomes Using Narrow-Diameter Implants for Single Upper Central Incisor Replacements

**DOI:** 10.3390/dj13040144

**Published:** 2025-03-26

**Authors:** Eduardo Anitua, Aitana Tarazona, Mohammad Hamdan Alkhraisat

**Affiliations:** 1 University Institute for Regenerative Medicine and Oral Implantology—UIRMI (UPV/EHU-Fundación Eduardo Anitua), 01007 Vitoria, Spain; aitana295@gmail.com (A.T.); dr.khraisat@gmail.com (M.H.A.); 2 BTI Biotechnology Institute, 01005 Vitoria, Spain; 3 Oral and Maxillofacial Surgery, Oral Medicine and Periodontics Department, Faculty of Dentistry, University of Jordan, Amman 11942, Jordan

**Keywords:** narrow-diameter implants, upper central incisor, esthetic zone, single-tooth implant, implant survival, marginal bone level

## Abstract

**Objectives:** The upper central incisors play a central role in esthetics, symmetry, and function. The purpose of this study is to evaluate the use of narrow-diameter implants (NDIs) for replacing single missing upper central incisors, addressing the gap in research regarding specific tooth types and their esthetic outcomes. **Methods:** This retrospective study included adult patients with a single missing upper central incisor replaced by NDIs. Exclusion criteria included patients who lost adjacent teeth during follow-up and patients with non-loaded implants. The primary outcome was peri-implant bone stability, while secondary outcomes included implant survival, technical complications, patient satisfaction, and esthetic evaluation using the Pink Esthetic Score (PES) and the White Esthetic Score (WES). Descriptive statistical analysis was performed. **Results:** A total of 64 NDIs were placed in 64 patients (mean age 55 ± 15 years; 40 females, 24 males). Implant diameters were 3.3 and 3.5 mm, with lengths ranging from 6.5 to 11.0 mm. The mean follow-up period was 42 ± 19 months. Marginal bone loss was −0.7 ± 0.9 mm mesially and −0.5 ± 0.7 mm distally. No implant failures were recorded. Esthetic outcomes were satisfactory, with a mean PES of 7.0 ± 2.6 and a mean WES of 7.9 ± 2.0. **Conclusions:** NDIs demonstrated high survival rates, marginal bone stability, and acceptable esthetic outcomes in the replacement of single upper central incisors.

## 1. Introduction

The upper central incisors play a central role in esthetics, symmetry, and function [1]. Replacing a single missing central incisor is always a challenge, as it is noticeable when smiling and it affects facial esthetics. The loss of a central incisor can have psychological implications for patients, often affecting their confidence and overall quality of life [2,3]. Since the upper central incisors receive occlusal forces, careful biomechanical planning is also necessary to prevent technical complications [4]. These considerations highlight the importance of achieving an optimal balance between esthetic and functional requirements to ensure the durability and effectiveness of implant-supported restorations. Meticulous treatment planning and execution are essential to achieve ideal gingival contour, symmetry, and papillary fill between the implant and adjacent teeth [5,6].

The anatomy of the central incisor region, along with the delicate balance between soft and hard tissues and the alveolar bone remodeling following tooth loss, can complicate implant placement [7,8,9]. Achieving proper positioning of the dental implant often requires alveolar ridge augmentation, which may involve bone and/or soft tissue procedures [10]. Standard-diameter implants (≥3.75 mm) may not be suitable in certain cases, necessitating the use of narrow-diameter implants [11,12].

Compared to standard-diameter implants, narrow-diameter implants (NDIs) have a reduced osseointegration surface and lower resistance to loading forces [13,14]. These limitations have prompted the development and implementation of clinical studies to evaluate the predictability and effectiveness of NDIs in oral rehabilitation. This is particularly important in cases where NDIs are selected to replace a single missing tooth [11,12,15,16]. Splinting multiple implants offers a distinct advantage by enhancing their capacity to withstand lateral forces and reducing the received mechanical stress [17,18].

Several systematic reviews and meta-analysis have evaluated the outcomes of NDIs as single-unit implants in the esthetic zone [11,12,15,16]. The diameters of the two-piece NDIs have been 2.9, 3.0, 3.25, and 3.3 mm [19,20,21,22,23,24,25,26,27,28,29]. These studies consistently indicate no significant differences in implant survival or success rates between NDIs and standard-diameter implants. The cumulative implant survival and success rates have been remarkably high, at 97.5% (95% confidence interval (CI): 94.5–98.9%) and 97.2% (95% CI: 94.2–98.7%), respectively, over a follow-up period of up to 36 months [12]. Mean marginal bone loss (MBL) after one year has been reported to be 0.44 mm (standard deviation (SD): 0.04; 95% CI: 0.36–0.52), with meta-analysis revealing a negligible mean difference of 0.02 mm (95% CI: −0.23 to 0.10) between the two implant types. Implant success rate, as reported by Telles et al., has ranged from 93.8% to 100% over a maximum follow-up of three years [15]. However, in a meta-analysis that included randomized and non-randomized clinical studies reported higher MBL in NDIs, likely due to narrower alveolar ridges in non-randomized studies [15]. Parize et al. have performed a fixed-effect meta-analysis, revealing no significant survival rate differences between NDIs and regular-diameter implants, with success rates ranging from 84.2% to 100% (mean: 95.2%) [16]. The analysis of the MBL has indicated a mean difference of 0.02 mm (95% CI: −0.21 to 0.25; *p* = 0.87), with no differences between both implants. Roccuzzo et al. have compared two diameters of narrow-diameter implants—2.9 mm and 3.3 mm—and have reported no statistically significant differences in the outcomes of marginal bone stability, mechanical complications, biological complications, and esthetics [19].

In these studies, narrow-diameter implants have been utilized in both the anterior and posterior regions of the maxilla and mandible, making it challenging to provide specific recommendations for a particular tooth type [11,12,15,16]. Additionally, there is a need for more studies that evaluate not only functional outcomes but also esthetic results [16]. Achieving optimal results in cases of tissue destruction caused by dental disease often requires multiple clinical procedures. However, in clinical practice, patients may decline treatment plans involving extensive regenerative and restorative procedures. In such cases, a compromise is necessary while ensuring patient satisfaction and long-term stability. This study presents the outcomes of this approach. To address this gap, this retrospective study has been designed to assess the use of narrow-diameter implants for replacing a single missing upper central incisor. The aims of this study have been to evaluate radiographic changes at the level of the crestal bone, assess the survival rate of narrow implants, and evaluate the esthetic outcome of the restorations.

## 2. Materials and Methods

This retrospective clinical study adhered to the STROBE (Strengthening the Reporting of Observational Studies in Epidemiology) guidelines for observational studies [30] (Supplementary Material). All procedures complied with the ethical principles outlines in the Declaration of Helsinki (1964) for research involving humans and with the standards set by institutional and national research committees. This study was part of a broader study assessing narrow-diameter implants, approved by the Ethics Committee of the Araba University Hospital (FIBEA-01-ER-23-Estrechos) on 30 July 2024.

An anonymized database was used to select patients for inclusion. The inclusion criteria were as follows: adult patients (age ≥ 18 years) with a single missing upper central incisor replaced by a single-unit narrow-diameter implant (diameter ≤ 3.5 mm), where the definitive crown had been delivered and peri-apical radiographs were available at the time of implant loading and at a subsequent follow-up. The exclusion criteria included the loss of adjacent teeth during the follow-up period and non-loaded implants.

### 2.1. Surgical Procedure

Preoperative assessment included cone beam computed tomography (CBCT) (NewTom, Imola, Italy) to evaluate bone quality and quantity at the implant site, as well as the height and width of the crestal bone. Measurements and surgical planning were conducted using BTI Scan software (BTI Scan IV; BTI Biotechnology Institute, Vitoria, Spain).

Plasma rich in growth factors (PRGFs) was used in all surgeries. For that, peripheral blood was drawn from each patient into 9 mL collection tubes containing 3.8% sodium citrate (KMU 15, BTI Biotechnology Institute, Vitoria, Spain). The blood was centrifuged at room temperature for 8 min to separate components. The plasma column was divided into two fractions: Fraction 1 (F1), used to create fibrin membranes, and Fraction 2 (F2), with a higher platelet concentration, used to regenerate [31]. To activate the platelets and the coagulation cascade, 20 µL of 10% calcium chloride (BTI Biotechnology Institute, Vitoria, Spain) was added per 1 mL of PRGFs.

Patients received 2 g of amoxicillin and 1 g of paracetamol 60 min before surgery as prophylactic medication. Procedures were performed under sedation and local infiltration anesthesia. Intrasulcular incisions with full-thickness flap elevation were made, followed by bone drilling using the biological drilling technique [32,33]. The initial drill operated at 800 rpm with saline irrigation, while subsequent drills, at 125 rpm, were used without irrigation. Autologous bone collected during drilling was mixed with liquid F2 for subsequent use. All implants (UnicCa^®^, BTI Biotechnology Institute, Vitoria, Spain), with body diameters of 3.3 mm or 3.5 mm and prosthetic platforms of 3.5 mm or 4.1 mm, were placed yuxtacrestally by the same surgical team. Where necessary, the labial alveolar bone plate was overcorrected using a bone graft combined with activated F2 in a 2:1 (*w*/*v*) ratio. The graft material consisted of autologous bone, anorganic bovine bone, or a mixture of the two. A fibrin membrane derived from F1 was used to cover the surgical area before flap closure.

Postoperative instructions, including oral hygiene recommendations, were provided to ensure cleanliness and care of the operated area.

### 2.2. Prosthetic Rehabilitation

All patients included in the study were restored using the same prosthetic concept, employing an intermediate abutment (UNIT^®^, BTI Biotechnology Institute, Vitoria, Spain). The definitive restorations included single-unit screw-retained prostheses. The restorations were fabricated from IPS e.max^®^ (Ivoclar, Schaan, Liechtenstein), a monolithic lithium disilicate ceramic, by the same laboratory using the same technique for all cases.

### 2.3. Study Variables

The primary variable of the study was peri-implant bone stability. Secondary variables included implant survival, technical complications, patient satisfaction, and esthetic evaluated using the Pink Esthetic Score (PES) and the White Esthetic Score (WES). In the absence of natural unrestored reference tooth, previous clinical pictures or digital records were used. Additionally, clinically relevant variables related to the patient (gender and age), the surgery (need for additional surgical techniques), and the implant (diameter and length) were also assessed.

**Figure 1:**
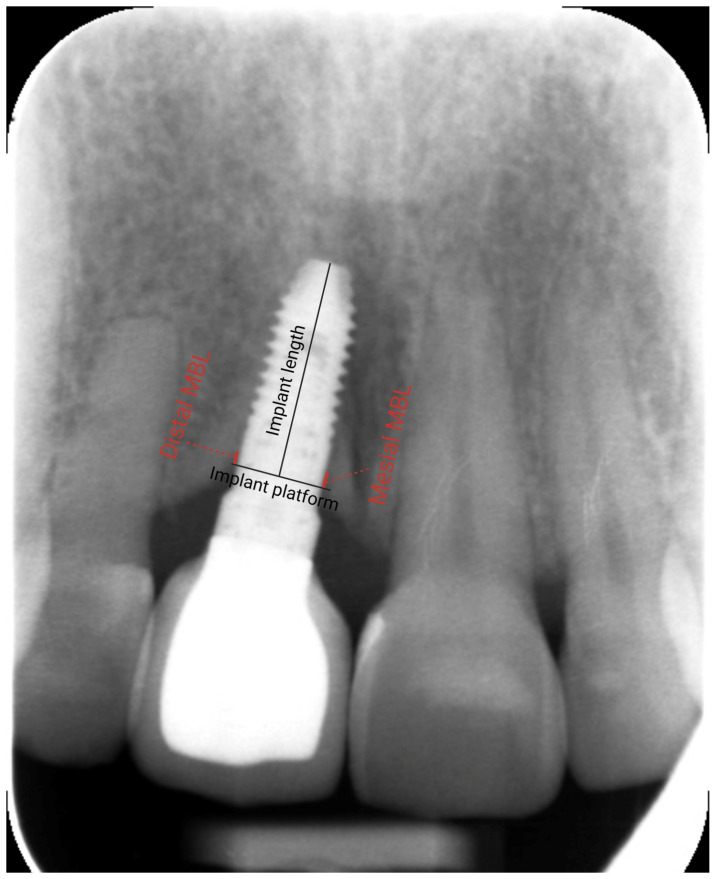
Periapical radiograph showing the measurement of marginal bone level. The known implant length was used to calibrate the radiographic measurements. Then, the mesial and distal marginal bone levels (MBLs) were measured.

To evaluate marginal bone stability, radiographic examinations were performed using standardized digital periapical radiographs (Figure 1). Peri-implant bone levels were measured using specialized software (DIGORA^®^ for Windows 2.9.113.490, Soredex, Milwaukee, WI, USA) at two time points: at the time of implant loading and on the most recent radiograph available at the time of the study. The measurements were calibrated based on the known implant length, and the distance between the implant shoulder and the most coronal bone-to-implant contact (mesially and distally) was recorded. Positive values indicated that the bone level was above the implant platform, while negative values signified that the bone level was below the implant platform. Changes in marginal bone levels were calculated as the difference between the bone level at loading and the most recent recorded level.

Technical complications (screw loosening, screw fracture, and chipping, among others) survival of the crown were assessed. For esthetics, intraoral photographs of all patients were taken, clearly capturing the soft tissues and implant-supported restorations, including the tooth under study and the adjacent teeth. Pink Esthetic Score (PES): The PES was used to evaluate seven variables: mesial papilla, distal papilla, soft tissue level, soft tissue contour, alveolar process deficiency, soft tissue color, and soft tissue texture. Each variable was scored from 0 to 2, where 0 represented the worst outcome and 2 represented the best outcome. The highest possible score was 14, indicating perfect peri-implant soft tissues. A threshold of 8 was considered clinically acceptable, while a score of 12 or more was deemed nearly perfect peri-implant soft tissues, as described by Fürhauser et al. [34]. White Esthetic Score (WES): The WES was used to assess five variables: overall tooth shape, tooth contour, tooth color (hue and value), surface texture, and translucency. Each variable was scored from 0 to 2, with 0 indicating the worst outcome and 2 indicating the best outcome. The implant-supported tooth was compared with the contralateral reference tooth to evaluate white esthetics. A maximum score of 10 was awarded for the best replication of the contralateral tooth. Thresholds for clinically acceptable or nearly perfect implant-supported crowns were set to be 6 and 9, respectively.

### 2.4. Statistical Analysis

The statistical analysis was conducted using specialized software [SPSS Statistics version 15 (IBM, Armonk, NY, USA)]. Categorical variables were expressed as absolute and relative frequencies. Continuous variables were mean and standard deviation, and range.

## 3. Results

The database included 1243 implants that had been placed in the upper central incisor; however, 64 implants supported single crowns. This study evaluated 64 narrow-diameter dental implants placed in 64 patients to replace a single missing central incisor. All the implants were placed between 2016 and 2022. The mean age of the participants was 55 ± 15 years (range: 18–85), comprising 40 females and 24 males. All the patients lost their central incisor due to periodontal disease. Data description according to the patients’ sex is provided in the Supplementary Material.

The narrow dental implants were 3.3 and 3.5 mm in diameter (Table 1) and their lengths varied between 6.5 and 11.0 mm, with 7.5 and 8.5 mm being the most common lengths.

At the implant site, the width of the alveolar bone at crest was 5.6 ± 1.5 mm. Bone type II was the most frequent (51 implants), and the bone density was 741 ± 151 units. The implants were placed with a mean insertion torque of 28 ± 15 Ncm. However, most implants (49 out of 64) required overcorrection of the vestibular plate (Table 2).

All the implants were connected to intermediate definitive abutment for screw-retained single crown. The length of the abutments varied from 1.5 to 3.5 mm, with 2 and 2.5 mm being the most frequently used (27 cases) (Table 2).

The mean follow-up period was 42 ± 19 months. Marginal bone level was evaluated at loading and at the last follow-up. At loading, the mesial marginal bone level was 0.7 ± 1.0 mm, and the distal marginal bone level was 0.4 ± 0.9 mm. By the last follow-up, the mesial and distal marginal bone levels were 0.0 ± 0.9 mm −0.1 ± 0.6 mm, respectively. Thus, the implants had a marginal bone loss of −0.7 ± 0.9 mm (mesial) and −0.5 ± 0.7 mm (distal). No implant failures were recorded during the follow-up period.

The esthetic outcomes were satisfactory, with a mean Pink Esthetic Score of 7.0 ± 2.6 and a mean White Esthetic Score of 7.9 ± 2.0 (Table 3). Few technical complications were reported. Four cases required a change of the intermediate abutment, one case experienced pressure on the peri-implant mucosa, and two cases had prosthetic screw loosening (Table 4).

**Figure 2:**
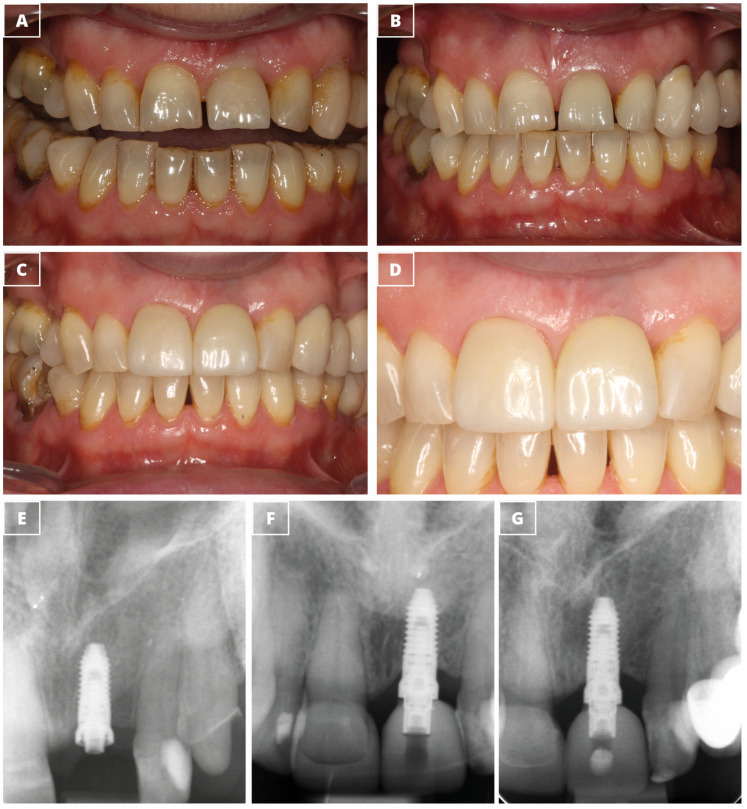
Clinical case treated by a single-unit narrow-diameter implant. (**A**) Clinical picture showing the baseline condition with the diagnosis of hopeless tooth #2.1 due to endo-periodontal disease. (**B**) The provisional prosthesis. (**C**) The definitive prosthesis. (**D**) The definitive prosthesis at higher magnification. (**E**) Periapical radiograph showing the insertion of the 3.5 mm × 8.5 mm dental implant. (**F**) Periapical radiograph at the implant loading. (**G**) Periapical radiograph after three years of implant insertion.

**Figure 3:**
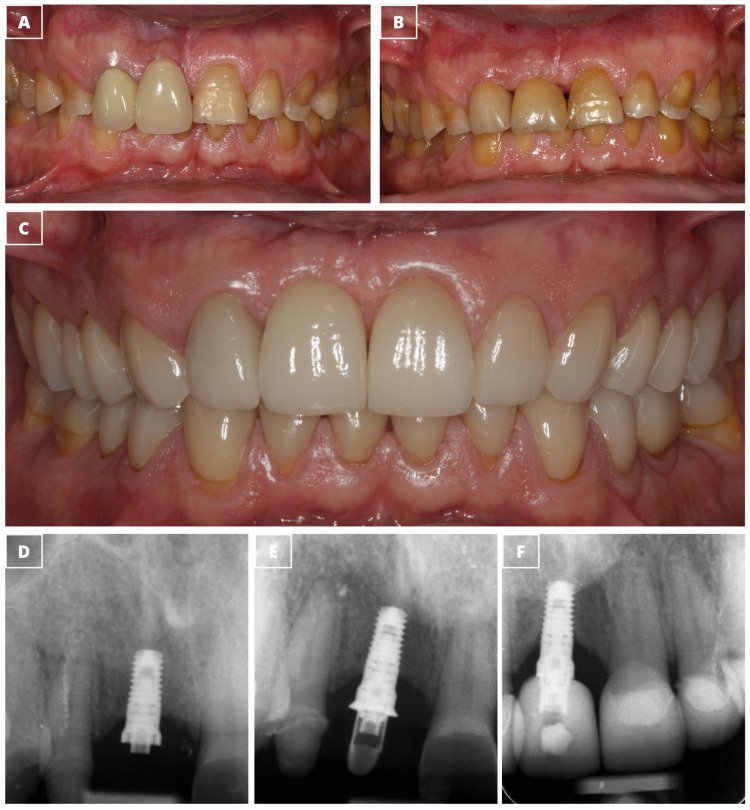
Clinical case treated by a single-unit narrow-diameter implant. (**A**) Clinical picture showing the baseline condition with missing #1.1 replaced by a fixed prosthesis. (**B**) The provisional prosthesis. (**C**) The definitive prosthesis. (**D**) Periapical radiograph showing the insertion of a 3.3 mm × 7.5 mm dental implant. (**E**) Periapical radiograph at the implant loading. (**F**) Periapical radiograph after four years of implant insertion.

Figure 2 and Figure 3 show two clinical cases that were treated with narrow-diameter implants.

## 4. Discussion

Meticulous planning of the implant position mesio-distally, corono-apically and oro-facially is of paramount importance when replacing a single missing upper central incisor with a dental implant [35,36]. The ITI consensus in 2004 defined a comfort zone of implant positioning in these three dimensions to avoid implant malpositioning and to minimize the risk of esthetic complications [35,36]. Adhering to these guidelines may impose constraints on the size of the dental implants, making narrow-diameter implants a viable and suitable option [36].

Narrow-diameter implants, initially used as transitional implants, are now being employed as definitive implants to support single crowns, partial prostheses, and complete prostheses in both the anterior and posterior regions of the maxilla and the mandible [16,37,38,39,40]. Narrow-diameter implants have a reduced osseointegration surface and lower resistance to loading forces than wider implants [13,14]. In the molar region, a single NDI is less reliable in supporting a single crown than standard implants or two NDIs [41]. Thus, it is important to take measures that enhance the biomechanical situation of the NDIs, aiming to reduce the loads received by the NDIs [38,39,42]. For example, splinting the implant together and reducing occlusal table and cusp inclination would enhance force distribution and reduce the lateral forces [17,18]. Taking these measures would make NDIs a reliable alternative in cases of horizontal atrophy, even in posterior sectors [42,43,44]. Some reports have indicated narrow-diameter implants as a risk factor for implant fracture, particularly in posterior regions and single restorations [45,46,47]. However, implant fracture is multifactorial where patient factors, implant design, prosthesis design, and marginal bone loss play a role [45]. Current evidence from several systematic reviews supports their clinical reliability [11,12,15,16]. The use of NDIs would minimize the need for alveolar ridge augmentation, thus reducing the number of surgical interventions required to restore prosthetically the clinical case [38,39].

In a study by Zhang et al. [48], NDIs (3.5 mm in diameter) have been compared with standard-diameter implants (4.3 mm in diameter) combined with lateral bone augmentation. No implant loss has been observed in either group during a 3-year follow-up. However, patient satisfaction ratings have been significantly higher in the narrow-diameter implant group compared to the standard-diameter implant group with lateral bone augmentation. Additionally, the total cumulative cost of treatment per patient has been significantly lower in the group of 3.5 mm implants (USD 2849.60, 95% CI: USD 2726.80–2972.40) compared to the 4.3 mm implant group with bone augmentation (USD 3581.40, 95% CI: USD 3460.90–3701.90) [48].

Narrow-diameter implants are generally classified into two main designs: one-piece and two-piece dental implants [16,49]. One-piece dental implants provide limited prosthetic options [16]. In contrast, two-piece dental implants offer the advantage of reversibility, allowing for the adaptation of the intermediate component of the implant system to potential changes in the peri-implant tissue. This adaptability is particularly valuable as both implants and patients age, requiring clinicians to address changes in peri-implant soft tissues through minimally invasive approaches [50]. In this context, the use of screw-retained crowns further facilitates the handling of clinical changes and complications [51].

This study addresses the gap in evidence regarding the management of specific tooth type and the esthetic outcomes of NDIs. Specifically, single missing upper central incisors were replaced with single crowns supported by 3.3 mm or 3.5 mm diameter implants. The follow-up period was 42 months, during which none of the 64 implants failed. This high survival rate aligns with findings from several systematic reviews reporting similarly high implant survival rates [11,12,15,16]. In a meta-analysis by Zhang et al., survival rates of narrow-diameter implants have ranged from 93.8% to 100% [11]. Cao et al. reported an implant survival rate of 97.5% (95% CI: 94.5%–98.9%) [12]. Telles et al. have documented implant success rates between 93.8% and 100% over a maximum follow-up of three years [15]. Similarly, Parize et al. have reported a mean success rate of 95.2% (rang: 84.2% to 100%) [16]. Narrow-diameter implants can be manufactured from titanium or titanium–zirconium alloy. In this study, the implants were made from commercially pure titanium. According to Cao et al., in their study of narrow-diameter implants with follow-up periods of up to 36 months, no significant differences have been observed between commercially pure titanium implants and titanium–zirconium implants in terms of survival or success rates [12].

The mean marginal bone observed in this study was −0.7 ± 0.8 mm mesially and −0.5 ± 0.6 mm distally. These findings are in agreement with reported data in several studies [11,12,15,16]. Cao et al. have reported a mean marginal bone loss (MBL) of 0.44 mm (SD: 0.04, 95% CI: 0.36–0.52) after one year of function [12]. Similarly, other studies have reported a mean marginal bone loss between 0.4 and 0.58 mm [15,52,53,54,55]. However, a higher MBL of 1.62 mm (SD: 0.61 mm) has been reported by Zarone et al. [56].

The use of definitive intermediate abutment may have favored the marginal bone stability around the NDIs. Studies suggest that intermediate abutments of at least 2 mm of height significantly minimizes the marginal bone loss [57,58]. Additionally, employing the “one abutment one time” protocol would also enhance the stability of marginal bone by avoiding repeated abutment connection/disconnection and thus the apical migration of the connective tissue [59,60]. In a histological human study, maintaining the definitive abutment without disconnection was identified as a factor that minimized trauma to peri-implant tissues and contributed to the prevention of marginal bone loss [61]. This approach would also reduce inflammation in the peri-implant tissues [62]. Furthermore, the use of original components would enhance the seal quality at the abutment–implant interface. A hermetic seal at this interface maintains peri-implant bone stability, minimizes stress, and prevents microorganism colonization [63,64,65,66]. Achieving this seal depends on precise machining of intermediate abutments, internal connection implants, and optimal screw preload to ensure proper fit, stress distribution, and resistance to loosening [67,68].

Esthetics is a key factor in the oral rehabilitation of the anterior maxillary region, where the upper central incisors play a pivotal role. Achieving optimal and stable esthetic outcomes depends on adequate treatment planning and execution. Proper 3D implant positioning allows for an optimal configuration of the esthetic zones in the transition of the prosthetic restoration from the implant platform to the oral cavity through the free gingival margin [69]. A soft tissues with a thickness of 2–3 mm is required to maintain a stable marginal bone level and to camouflage the underlying abutment [70,71]. It is important to adapt the design of the restoration to support long-term stability of the soft tissue, regarding shape and position [69]. Patients are mainly concerned by discoloration and recession of the buccal gingiva [20]. Additionally, they place significant importance on dentogingival esthetic parameters, including dental, gingival, and occlusal features, that are largely influenced by the proportion, shape, and position of the central incisors, as well as their relationship with adjacent dental structures [1]. To objectively evaluate esthetics, several indices have been used in the clinical practice such as the PES and the WES [72]. While esthetic outcomes assessed using the PES and WES may not always correlate with patients’ subjective perceptions, these indices remain valuable tools for monitoring implant performance [73,74]. In this study, the mean PES score was 7 and the mean WES score was 8, reflecting an acceptable result. Notably, all patients expressed satisfaction with their prosthesis. The goal of any treatment is to ensure patient satisfaction and long-term stability [75]. In this study, all the patients have lost the upper central incisor as a consequence of periodontal disease, indicating advanced periodontal disease that would influence negatively the PES score [76]. Tissue destruction will lead to the need for multidisciplinary clinical procedures to compensate for tissue loss. The increased number of procedures would affect time and cost and thus the willingness of the patients to accept them. The expectations of the patients would assist the clinician in adapting the treatment plan [77]. The study outcomes have indicated that the described clinical procedure, followed in this study, has resulted in patient satisfaction and long-term stability. According to Pieri et al. (2014), mean scores of 6.96 (±0.92) for the Pink Esthetic Score and 7.1 (±1.09) for the White Esthetic Score were reported [21]. No significant differences were observed in either parameter when comparing baseline (crown placement) with the 3-year follow-up [21]. In another study, a significant improvement in the Pink Esthetic Score has been observed, increasing from 6.3 (±0.4) at baseline to 10.5 (±2.5) after a 1-year follow-up [29]. Zhang et al. have found that soft tissue dehiscence occurred more frequently in regular-diameter implants combined with alveolar bone augmentation compared to narrow-diameter implants [11]. Additionally, the narrow-diameter implants would achieve more rapid esthetic improvements. The acceptable esthetic results observed in this study could be influenced by the inclusion of single missing central incisors. The formation of interdental papilla is more likely between natural tooth and implant rather than two adjacent implants [16,52]. Furthermore, the stability of the marginal alveolar bone likely contributes to the stability of the soft tissues around the dental implants [16]. Additionally, the use of narrow implants would provide additional space for the peri-implant tissue [15].

Abutment screw loosening is one of the most commonly reported prosthetic complications in clinical studies on NDIs. This issue may result from various factors, including component misfit, inadequate tightening, screw settling, suboptimal screw design, or excessive loading [78]. In our study, two events of screw loosening, which were resolved by applying the correct torque to the screw, were observed. Thus, screw loosening in this case could be most probably related to inadequate tightening torque.

This study has several limitations, including the absence of a control group and a relatively short follow-up period. As a retrospective analysis of previously collected data, this study inherently depends on the availability and quality of the database, which poses the risk of missing or incomplete information. This study has not assessed the changes in the MBL at different time points in the follow-up; instead, the MBL has been assessed at baseline and in the last available radiograph. Patient-reported outcomes have not been assessed due to the retrospective nature of the study. However, the retrospective design provides insights into real-world scenarios and daily clinical practice. It is important to interpret the results with caution, as the limitations may affect the generalizability of the findings. Furthermore, this study specifically assessed cases of single missing upper central incisors, where the presence of adjacent natural dentition likely contributed to the stability of peri-implant tissues.

## 5. Conclusions

The use of narrow-diameter dental implants to support single crowns replacing a single missing upper central incisor has demonstrated high implant survival rates, marginal bone stability, patient satisfaction, and acceptable esthetic outcomes. Nevertheless, more studies are needed to assess the use of narrow-diameter implants to replace a single missing central incisor, as it plays a central role in esthetics.

## Figures and Tables

**Table 1:** The lengths and diameters of the dental implants placed to replace a missing central incisor.

		Length (mm)					Total
		6.5	7.5	8.5	10.0	11.0	
Diameter (mm)	3.3	5	10	10	2	0	27
	3.5	5	14	14	3	1	37
Total		10	24	24	5	1	64

**Table 2:** Surgical-related parameters.

Variable		Value
Width of the alveolar bone at crest (mean ± SD)		5.6 ± 1.5
Bone type (number)	Type I	6
	Type II	51
	Type III	7
Bone density (A.U) (mean ± SD)		741 ± 152
Insertion torque (N.cm) (mean ± SD)		28 ± 15
Overcorrection of the vestibular plate	Yes	49
	No	15
Intermediate abutment lengths	1.5 mm	3
	2 mm	27
	2.5 mm	21
	3.0 mm	11
	3.5 mm	2

SD: standard deviation.

**Table 3:** Dental-implant-related variables.

Variable		Value
Follow-up time (months)		42 ± 19
Mesial MBL (mm) (mean ± SD)	Loading	0.7 ± 1.0
Distal MBL (mm) (mean ± SD)		0.4 ± 0.9
Mesial MBL (mm) (mean ± SD)	Last radiograph	0.0 ± 0.9
Distal MBL (mm) (mean ± SD)		−0.1 ± 0.6
Variation in MBL–mesial (mm) (mean ± SD)		−0.7 ± 0.9
Variation in MBL–distal (mm) (mean ± SD)		−0.5 ± 0.7
Implant failure (frequency)		0
Pink Esthetic Score		7.0 ± 2.6
White Esthetic Score		7.9 ± 2.0

MBL: marginal bone level; SD: standard deviation.

**Table 4:** Technical complications and their frequency.

Variable	Value
Change the intermediate abutment	4
Pressure on peri-implant mucosa	1
Screw loosening	2

## Data Availability

The datasets used and/or analyzed during the current study are available from the corresponding author upon reasonable request.

## Supplementary Materials

The following supporting information can be downloaded at https://www.mdpi.com/article/10.3390/dj13040144/s1. Table S1: STROB checklist and data described according to patients’ sex.
